# Mycotic infection as a risk factor for COVID-19: A meta-analysis

**DOI:** 10.3389/fpubh.2022.943234

**Published:** 2022-09-07

**Authors:** Anlin Liu, Zhengtu Li, Guansheng Su, Ya Li, Yuzhuo Zhang, Jinkai Liang, Xiaoxue Cheng, Xidong Wang, Yongming Li, Feng Ye

**Affiliations:** ^1^State Key Laboratory of Respiratory Disease, National Clinical Research Center for Respiratory Disease, Guangzhou Institute of Respiratory Health, The First Affiliated Hospital of Guangzhou Medical University, Guangzhou, China; ^2^Nanshan School of Guangzhou Medical University, Guangzhou, China

**Keywords:** corona virus disease (COVID-19), mycotic infection, risk factor, meta-analysis, subgroup analysis

## Abstract

More than 405 million people have contracted coronavirus disease 2019 (COVID-19) worldwide, and mycotic infection may be related to COVID-19 development. There are a large number of reports showing that COVID-19 patients with mycotic infection have an increased risk of mortality. However, whether mycotic infection can be considered a risk factor for COVID-19 remains unknown. We searched the PubMed and Web of Science databases for studies published from inception to December 27, 2021. Pooled effect sizes were calculated according to a random-effects model or fixed-effect model, depending on heterogeneity. We also performed subgroup analyses to identify differences in mortality rates between continents and fungal species. A total of 20 articles were included in this study. Compared with the controls, patients with mycotic infection had an odds ratio (OR) of 2.69 [95% confidence interval (CI): 2.22–3.26] for mortality and an OR of 2.28 (95% CI: 1.65–3.16) for renal replacement therapy (RRT). We also conducted two subgroup analyses based on continent and fungal species, and we found that Europe and Asia had the highest ORs, while Candida was the most dangerous strain of fungi. We performed Egger's test and Begg's test to evaluate the publication bias of the included articles, and the *p*-value was 0.423, which indicated no significant bias. Mycotic infection can be regarded as a risk factor for COVID-19, and decision makers should be made aware of this risk.

## Introduction

Coronavirus disease 2019 (COVID-19), caused by severe acute respiratory syndrome coronavirus 2 (SARS-CoV-2), can result in severe acute respiratory syndrome; COVID-19 has rapidly been spreading worldwide for more than 2 years, resulting in more than 405 million individuals contracting the disease (1). It can not only cause damage to the respiratory system but also influence the cardiovascular, digestive and nervous systems, resulting in multiple organ dysfunction syndrome (MODS) (2). As we all know that fungi are ubiquitous in our living environment, and although a large number of species exist in our environment, few species of fungi can cause fungal infection, and the incidence of fungal infection is low despite exposure, especially among those with a normal immune system (3–5). Pulmonary aspergillosis is hard to diagnosis and treat, which shows unspecific clinical presentations and not fully identical image results when occur (6). Meanwhile, invasive pulmonary aspergillosis (IPA) is a sever disease that the mortality of IPA exceeds 50% in neutropenic patients and reaches 90% in hematopoietic stem-cell trans-plantation (HSCT) recipients (7). As for Candida, invasive Candidiasis is the most frequent fungal infection related to health care service, which is also associated with higher burden like high mortality, morbidity and cost (8). Attributable mortality reaches nearly 40% in America while it's the most common cause of blood stream infection in US health centers (9). Moreover, Mucormycosis is a rare but aggressive fungal disease that mainly occur in people with underlying disease, and it's challenging for us to diagnosis it, even if the growth of microbiology tools is at a fast pace (10). However, when fungal infection and COVID-19 co-occur, health risks increase. Rawson et al. (11) reported that 8% of patients developed bacterial or fungal coinfection during hospital admission, especially patients admitted to the intensive care unit (ICU), and the prevalence of invasive fungal infection was 1.6%. The reported incidence rate of COVID-19-associated pulmonary aspergillosis (CAPA) ranged between <5 and >30% in different case series (12–14), and according to Singh et al. (15), the mortality rate of CAPA can reach 51.2%, which is egregious and concerning. Treatment for CAPA patients is also a challenge, as reported by Chong and Neu (16), who conducted a systematic analysis and found that the pooled mortality of CAPA patients was 48.4%, even when treated with antifungals. Moreover, anti-COVID treatments can exacerbate respiratory conditions in patients who possibly suffer from IPA (14), especially those treated with corticosteroids (12). According to Lai and Wu (17), the incidence rate of IPA in COVID-19 patients ranged from 19.6 to 33.3%. However, there is a lack of high-quality evidence to support decision making regarding bacterial and fungal infections, and the reported difficulties in distinguishing bacterial and fungal infections and COVID-19 are alarming (18, 19).

Few articles on the possible connection between IPA or CAPA and COVID-19 have been published, but their association with COVID-19 remains a vital issue to elucidate. One article previously investigated the pooled mortality rate in patients with CAPA and investigated the OR for mortality in CAPA plus COVID-19 patients compared with only COVID-19 patients (15). However, the author did not define mycotic infection as a risk factor. Another systemic analysis published in March also reported the pooled mortality and total incidence rates of CAPA in ICU patients (20). Chong and Neu (16) reported the detailed characteristics of CAPA patients in a systematic review, while Apostolopoulou et al. (12) investigated the epidemiology, pathogenesis, clinical manifestations, diagnosis and treatment of and risk factors for CAPA. In conclusion, further investigation is needed to define fungal infection as a risk factor for COVID-19. Therefore, we conducted this meta-analysis to define fungal infection, especially pulmonary aspergillus infection, as a risk factor for COVID-19.

The present situation suggests the need for further investigations into COVID-19 and fungal coinfection and potential risk factors. Therefore, we conducted this meta-analysis to elucidate whether fungal infection is a risk factor for COVID-19 and provide decision makers with evidence that patients with COVID-19 and mycotic coinfection (CMI) need additional attention.

## Methods

### Search strategy

Two investigators (LY and SG) independently searched the Web of Science and PubMed databases for studies published from December 1, 2019, to December 27, 2021. These articles were freely available due to the ongoing COVID-19 situation. The search strategy was performed using Boolean operators with MeSH terms, such as “Pulmonary aspergillosis AND COVID- 19”, “Mucormycosis AND COVID-19”, “Candidiasis AND COVID-19” and “Fungal infection AND COVID-19”. Further details about the search strategy are presented in Supplementary methods. After the first search, we obtained 1,353 articles.

### Study selection

Two investigators (LZ and ZY) independently reviewed all potentially available manuscripts. Disagreements or uncertainties were resolved by a different group of investigators (CX and YF). The initial screen mainly focused on reviewing the abstract and titles, while the secondary screen included full-text review. In the initial screening stage, articles that were not COVID-19- or fungal infection-related were excluded, and in the second screening stage, we applied the inclusion and exclusion criteria to the articles.

The studies included in our systematic review and meta-analysis met the following inclusion criteria:

1. Case–control, cohort or cross-sectional study design;
2. Comparison of at least two groups, including COVID-19 patients without mycotic infection and COVID-19 patients with mycotic coinfection;
3. Inclusion of data on the detailed clinical characteristics of pooled patients, including mortality rates for each group.
4. Independence from other studies.

Exclusion criteria:

1. Other study designs, including case reports, case series and reviews;
2. Duplicate reports;
3. Inappropriate data collection methods or failure to provide detailed data on patient clinical characteristics.

### Data extraction

Two investigators (ZY and LY) independently evaluated the quality of all the identified manuscripts and extracted and entered the data for those that met the inclusion criteria. The authors identified and verified these articles independently, while disagreements were investigated by another group of investigators (CX and YF). We divided the articles evenly between researchers, and the following data were extracted: first author, year, country, study design: multi/single center, fungal species, mean age of the experimental group [±standard deviation (SD)], mean age of the control group (±SD), ICU length of stay, mean ICU length of stay of the control group, mean mortality, mortality in the control group, RRT rate, RRT rate in the control group.

### Quality assessment of the included studies

Two investigators (LA and LZ) independently assessed the quality of the included studies using the Newcastle–Ottawa quality assessment scale (NOS), and careful assessment of all the included manuscripts revealed that they all fulfilled the criteria for good quality. The detailed assessment results are shown in Supplementary Table 1. We also conducted Egger's test and Begg's test to evaluate publication bias of the included manuscripts.

### Statistical analysis

The primary outcome of this meta-analysis was the OR for COVID-19 in patients with mycotic disease (case group) compared with those without mycotic disease (control group). Subgroup analyses were performed according to continent and the fungal species causing infection, resulting in several subgroups. Heterogeneity within the pooled studies was evaluated using the *I*^2^ statistic (significant if *I*^2^ ≥ 50%); depending on whether there was heterogeneity between studies, a random-effects model was used. Publication bias was evaluated with Egger's test, Begg's test and a funnel graph. All statistical analyses were conducted using Stata/SE version 15.1 (Stata Corp LLC, College Station, TX, USA), and a *P*-value <0.05 was considered statistically significant.

## Results

### Literature search and general descriptions of the included studies

**Figure 1:**
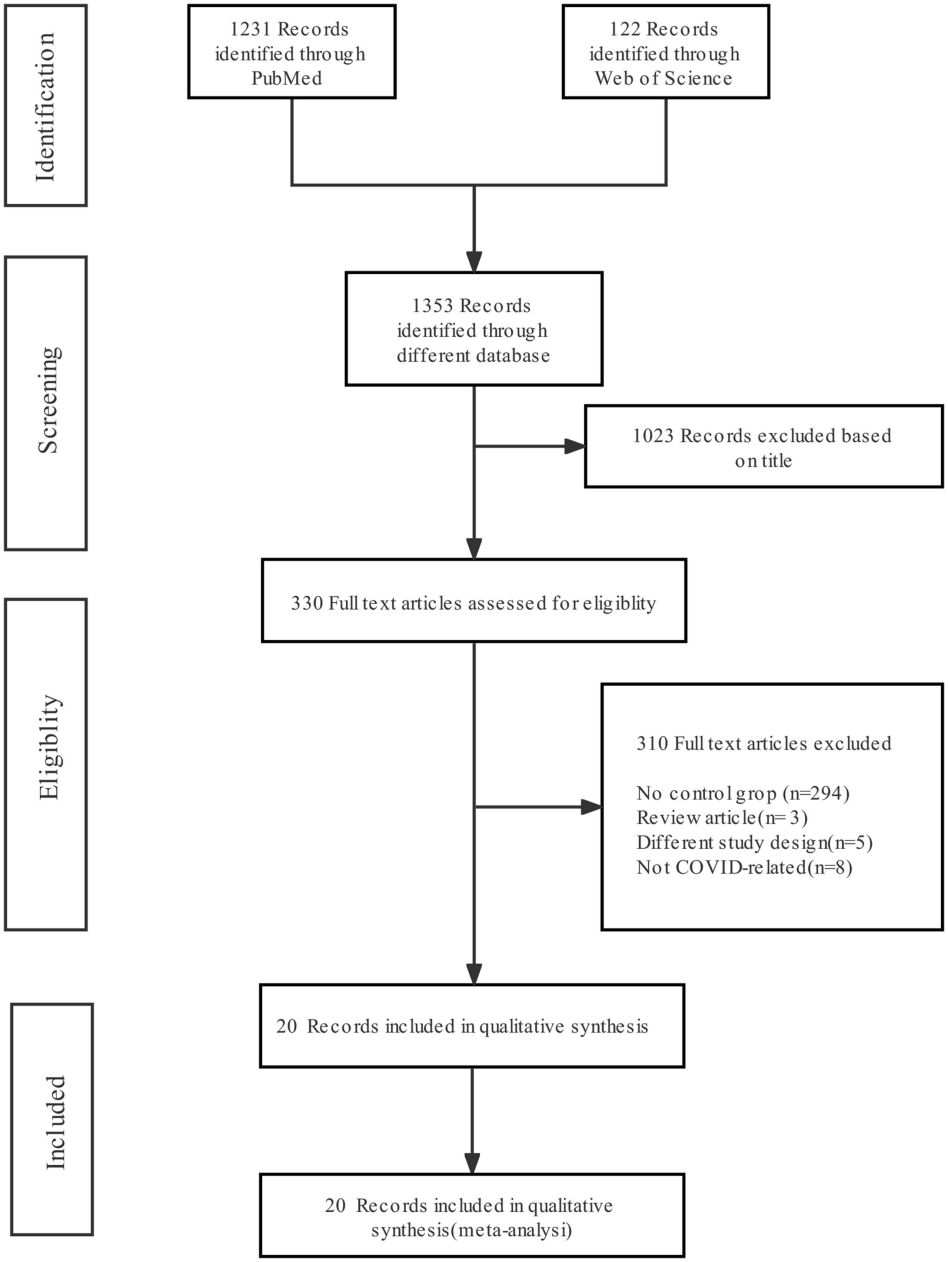
Preferred Reporting Items for Systematic Reviews and Meta-Analysis (PRISMA) flow diagram of the search results and article exclusion details.

Figure 1 shows the literature screening flow chart, which includes the number of articles identified in the databases (PubMed and Web of Science), the number of excluded studies and the reasons for exclusion. Of 1,353 abstracts identified during initial screening, 330 were selected for full-text review; 1,023 were excluded due to absence of the combination of “COVID-19” and “fungal infection”. After the full texts were investigated, 20 were included for analysis; the others were excluded for the following reasons: missing detailed clinical information about COVID-19 patients without fungal infection and missing mortality or death data.

The characteristics of the included studies are presented in Supplementary Table 2. In general, 3,128 patients were included in our study, 624 of whom suffered from both COVID-19 and pulmonary fungal infection. In our study pool, 294 patients developed aspergillosis, while 10 suffered from *Pneumocystis jirovecii* infection, and 116 suffered from candida infection. The majority of patients had Aspergillus infection, among whom 156 patients died. The detailed species of Candida.spp is shown in Supplementary Table 3.

All studies were published between 2020 and 2021, and the majority of the articles were based in Europe (two in Italy, three in the Netherlands, five in France and one each in Spain and Germany). The methodological quality of the included studies was satisfactory.

### Pooled OR for mortality and subgroup analysis based on continents and species of fungi

**Figure 2:**
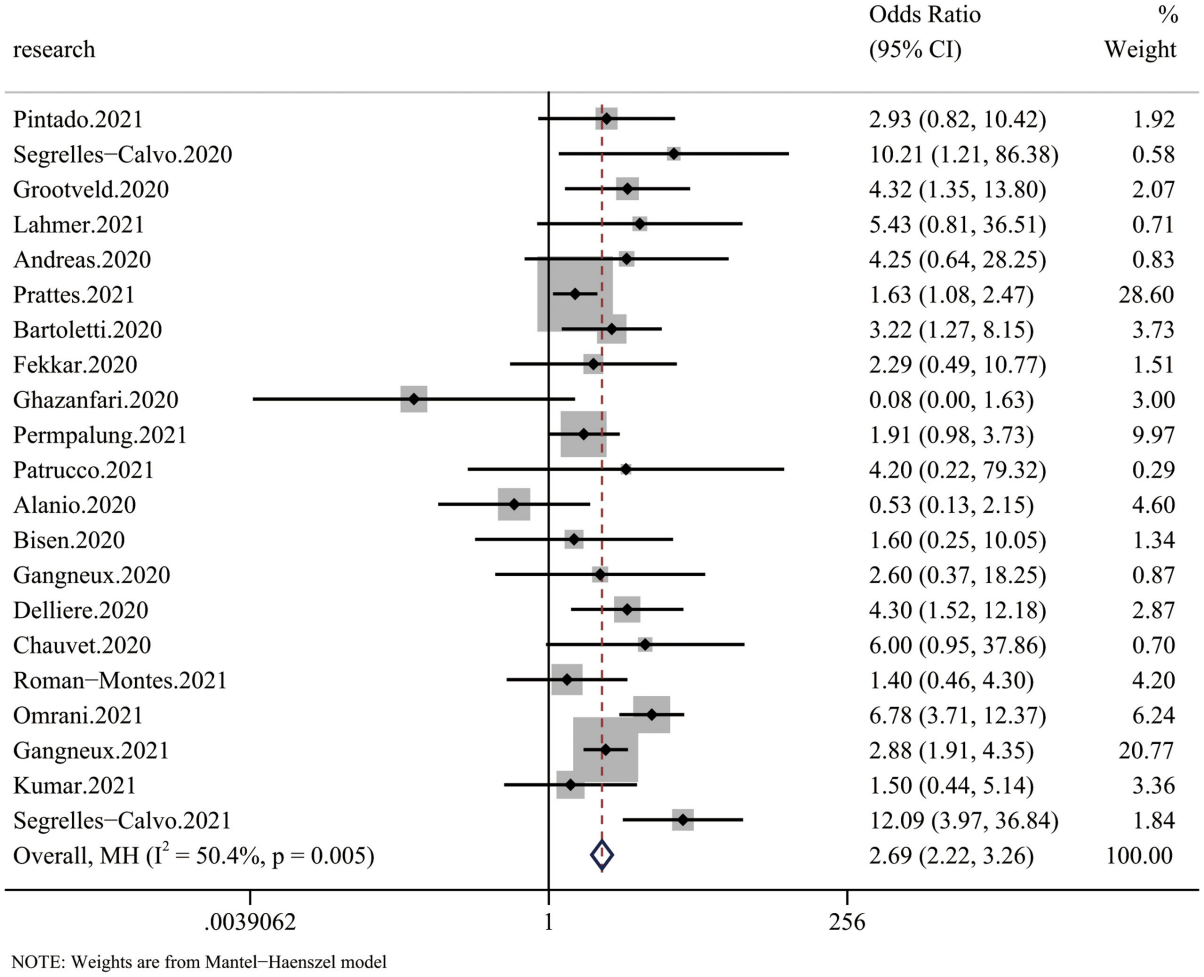
Forest plot of pooled mortality showing the ORs of the included articles (*n* = 20). The pooled OR for COVID-19 associated with mycotic infection (CMI) was 2.69 (95% CI: 2.26–3.33).

All 20 articles analyzed the relationship between COVID-19 and mycotic disease, and all of them reported detailed mortality data of the case group and the control group. Compared with that in non-CMI patients, the risk of fatal outcomes in CMI patients was higher (OR 2.69, 95% CI: 2.22–3.26), with significant between-study heterogeneity (*I*^2^ = 44.1%, *p* = 0.016) (Figure 2). The publication bias results are shown in **Figure 5**.

We also conducted two subgroup analyses to further characterize mortality. In the first subgroup analysis, we analyzed differences between geographic areas. We divided the included patients into the following groups: Latin America, Europe, Asia and North America. The reason why some articles were labeled “multicontinents” was that they did not provide detailed information about the included patients or did not match the outcome to each patient. In the other subgroup analysis, we divided patients by the species of fungi causing infection. Some articles did not provide information about the specific type of fungus, so we included those patients in the “unspecified fungi” subgroup, which is not discussed due to uncertainty about the details.

**Figure 3:**
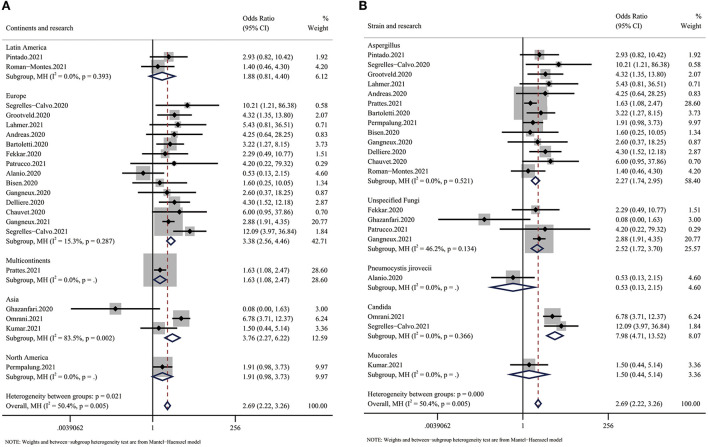
Forest plot showing the results of the subgroup analysis. **(A)** Forest plot of the OR for mortality in CMI patients compared with non-CMI patients on different continents. The OR for Europe was 3.38 (95% CI: 2.56–4.46), the OR for Asia was 3.76 (95% CI: 2.27–6.22), and the OR for Latin America was 1.88 (95% CI: 0.81–4.40). **(B)** Forest plot of the OR for mortality in COVID-19 patients infected with different species of fungi. The OR for COVID-19 in patients with Aspergillus infection was 2.27 (95% CI: 1.74–2.95), the OR for COVID-19 in patients with Candida infection was 7.98 (95% CI: 4.71–13.52), and the OR for COVID-19 in patients with unspecified fungal infection was 2.52 (95% CI: 1.72–3.70).

The subgroup analysis based on continent showed a large difference in mortality between different regions. Despite the small number of articles, we found that Latin America had the lowest OR for mortality (OR: 1.88, 95% CI: 0.81–4.40), while Europe and Asia had much higher ORs (EU: 3.38, Asia: 3.76) (Figure 3A). Additionally, we found that infections by different species of fungi were associated with different outcomes. Although data to calculate the pooled outcome were insufficient, Candida spp. infection, with an OR for mortality of 7.98 (95% CI: 4.71–13.52), seemed to be more dangerous than Aspergillus infection (OR 2.39, 95% CI: 1.83–3.12) (Figure 3B).

### RRT outcome

**Figure 4:**
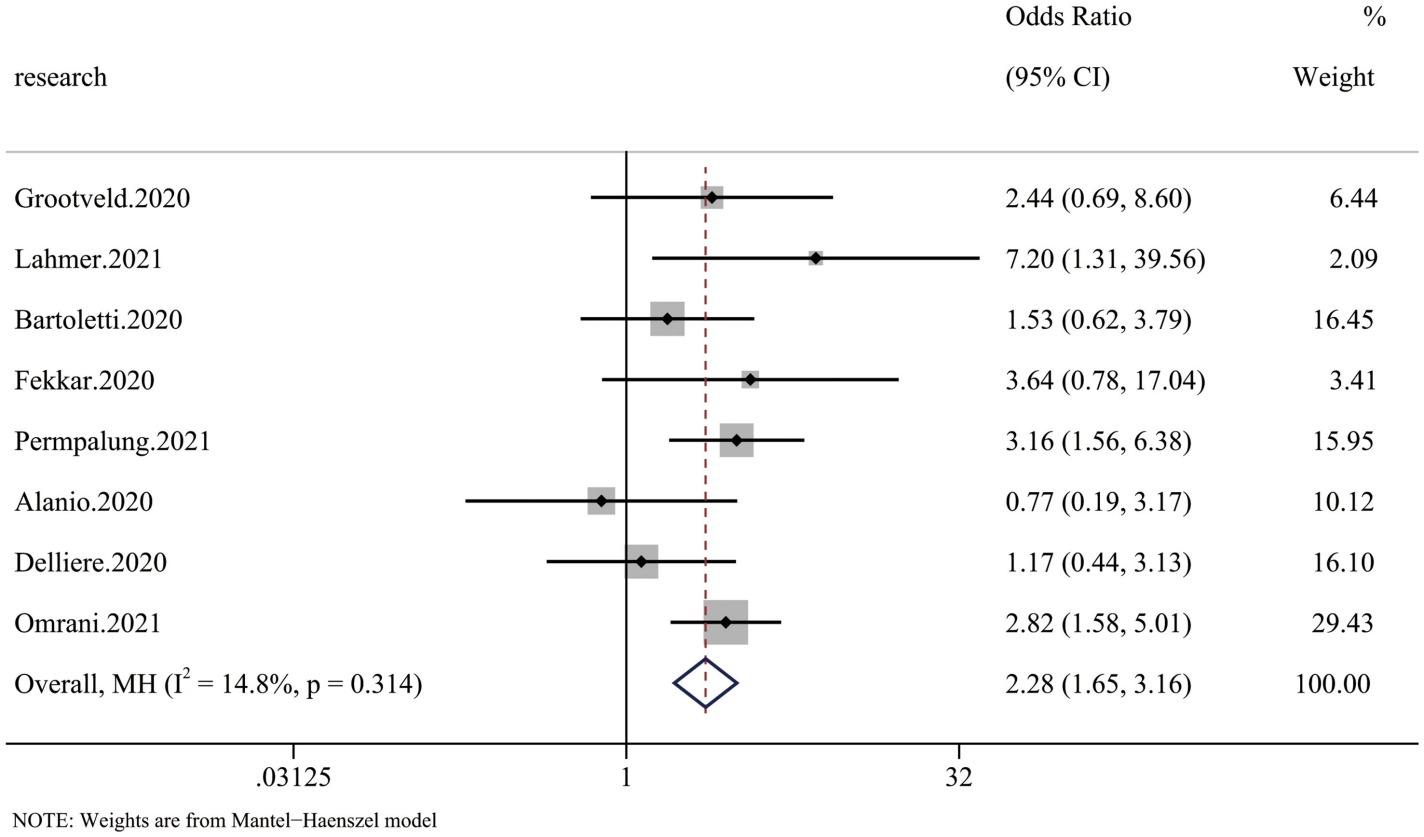
Forest plot of the pooled renal replacement therapy (RRT) rate. The OR for RRT in CMI patients compared with non-CMI patients was 2.28 (95% CI: 1.65–3.16).

Six of 20 articles investigated the relationship between COVID-19 and mycotic disease, and some included patients receiving continuous renal replacement therapy (CRRT), which we regarded as RRT. The pooled analysis showed that compared with patients without fungal infection, those with fungal infection had a significantly higher risk of requiring RRT (OR 2.28 95% CI: 1.65–3.16), with significant between-study heterogeneity (*I*^2^ = 14.8%, *p* = 0.314) (Figure 4).

### Bias control

**Figure 5:**
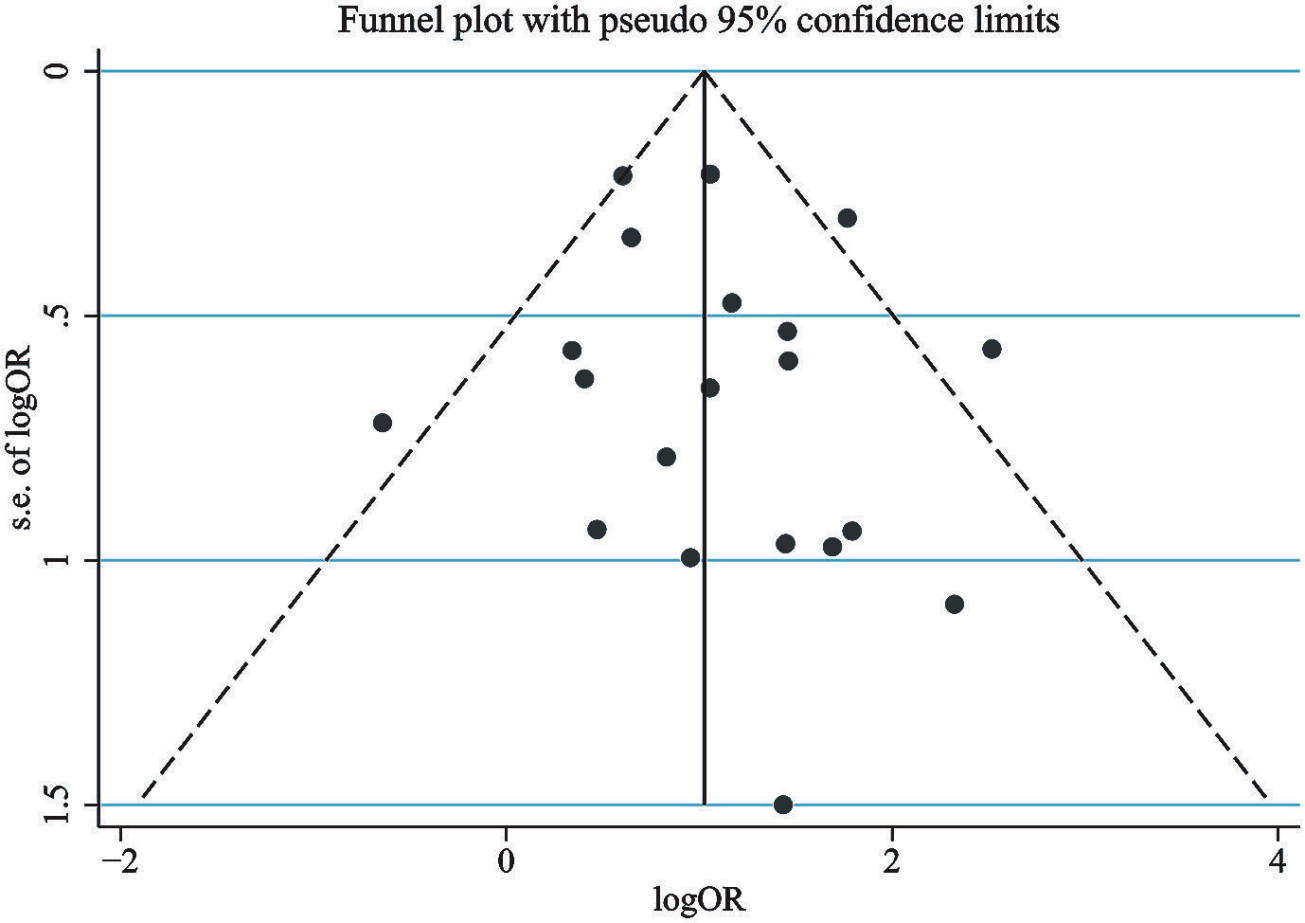
Funnel plot of the included manuscripts, showing no significant publication bias.

We conducted Egger's test and Begg's test to evaluate the publication bias of the included articles, and the *P*-value was 0.423, which indicated no significant publication bias in the pooled articles. The exact results of these tests are shown in Supplementary Figure 1. We also constructed a funnel plot to assess bias, and the results are shown in Figure 5.

## Discussion

We performed this meta-analysis to elucidate whether mycotic infection is a risk factor for COVID-19, and we found a 2.69-fold increased risk of mortality and a 2.28-fold higher RRT rate in CMI patients (Figures 2, 4). The risk of mortality was much higher in Europe and Asia than in Latin America (Figure 3A), which is partly due to more systematic medical services. Additional case–control studies based in Africa and South America are needed to indicate the overall situation. For the different species of fungi, we noticed that mortality associated with Candida coinfection was higher than that associated with Aspergillus coinfection (Figure 3B).

Articles have reported the OR for mortality in CMI patients compared with non-CMI patients. Singh et al. (15) reported a pooled OR of 2.77 (95% CI: 1.80, 4.25), and Chong et al. reported a pooled OR of 3.39 (95% CI: 1.97, 5.86) (21). As a result, we believe our result is accurate. Our data differ from the former articles in several aspects. Above all, we expanded the criteria for mycotic infection; therefore, we obtained more adequate data to perform our meta-analysis.

The reason we conducted this meta-analysis was that although articles reporting pooled ORs in CMI patients exist, few of them have identified mycotic infection as a risk factor for COVID-19. As the COVID-19 pandemic progresses, the incidence rate of COVID-19 and mycotic coinfection is increasing, and diagnosing COVID-19 is difficult due to its non-specific signs and clinical characteristics, especially when combined with mycotic infection. Additionally, they can result in similar clinical outcomes (17). Reports have indicated that mycotic infection increases the risk of mortality of COVID-19 patients, and we believe that fungal infection is dangerous to COVID-19 patients. The lack of articles defining mycotic infection as a risk factor for COVID-19 may lead to ignorance of the severity of CMI.

Interestingly, as a highly developed area, it was expected that Europe would respond better to mycotic coinfection, but the OR for Europe was one of the highest. The OR for Asia was the highest (3.76), which means that Asian countries need to place more emphasis on preventing CMI. Regarding the strains of fungi, the included articles mainly discussed the relationship between COVID-19 and a certain strain of fungi (12, 14–16, 20–22), while our result shows a much stronger relationship between mycotic infection and increased mortality in COVID-19 patients. In the current study, the number of pooled patients was much larger than those in previous studies, which may explain the difference in results of the increased risk of COVID-19 and mycotic coinfection. We found that COVID-19 patients infected with Candida had a higher mortality rate than those infected with Aspergillus. Segrelles-Calvo et al. (23) reported that the incidence rate of Candida infection in COVID-19 patients was 14.4%. Considering its incidence rate combined with its high OR for COVID-19, all decision makers should pay more attention to Candida coinfection. For Aspergillus, the incidence rate has been reported to range from <5 to >30%, which is also a substantial threat to COVID-19 patients, and colonization is more common than invasive disease. Although we found that Candida had a higher OR for mortality, the incidence rates of both Aspergillus and Candida infection were high, and it is a substantial challenge for medical professionals to identify, diagnose and treat CMI patients early (24).

RRT is a method of renal support for patients with acute kidney injury (AKI) (25); therefore, it can be regarded as a criterion for assessing severity. Yang et al. (26) reported that the rates of AKI and RRT were very high in COVID-19 patients, especially those admitted to the ICU (16.3% received RRT). We investigated the pooled RRT rate in CMI patients, and we found a significantly higher incidence rate of RRT in those with CMI than in those with only COVID-19, which indicated that mycotic infection may be associated with increased severity of COVID-19.

Unfortunately, the exact mechanism by which fungi increase the risk of mortality in COVID-19 patients is not yet clear, nor is it clear how COVID-19 increases the risk of fungal coinfection. However, authors have conducted investigations of this issue. Some articles have pointed out that the use of glucocorticoids (GCs), which may help control inflammation and reduce mortality in COVID-19 patients (27–29), may lead to immunocompromise, which results in an increased risk of coinfection (30). In addition, according to Kousha et al. (7), the most important risk factor for pulmonary aspergillosis is neutropenia, especially when absolute neutrophil counts are low, and there have been reports that neutrophil extracellular traps are related to cytokine storms, which may lead to hyperinflammation, lung injury, acute respiratory distress syndrome (ARDS) and death (31, 32); this may be a possible mechanism for the high incidence of COVID-19-related infections. Al-Tawfiq et al. (33) reported that patients who were diabetic and receiving corticosteroid therapy to control the severity of COVID-19 had higher fatality rates than their counterparts, complicating the pandemic scenario; this should raise awareness of the need to understand probable drawbacks of COVID-19 treatments. In conclusion, the fundamental reason for CMI is that COVID-19 induces an immunocompromised state (34). Therefore, it is essential to focus on coinfection during the treatment of COVID-19, especially in those on mechanical ventilation. The incidence of invasive aspergillosis can reach 30% in intubated patients, and some reports have reported incidence rates of 38%, which shows the high risk associated with intubation (35, 36).

In conclusion, we believe that COVID-19 is a risk factor for mycotic infection due to its high ORs for adverse outcomes, and decision makers should pay more attention to the risks of COVID-19 and mycotic coinfection. Some articles have tried to elucidate whether administering antifungals as a preventive measure against COVID-19 is useful. Hatzl et al. (37) found that the incidence rate was reduced, but the survival rate was not significantly changed. Whether we should use antifungals for preventive purposes remains unknown. Therefore, due to the increased risk of fatal outcomes and the uncertainty of administering antifungals prophylactically, measures should be taken to prevent patients from developing mycotic infection. Fungal infection patients should receive early antifungal treatment, which is related to increased disease severity and mortality in patients.

There are certain limitations to our investigation. It is not known whether our results are applicable to other ethnicities, as our data was mainly from Europe. In addition, it has been reported that the mortality rate in patients with both COVID-19 and *Candida albicans* infection is 40% (38), and 30.7% of patients develop rhino-orbital mucormycosis (39). However, due to the small number of studies that met our criteria, we mainly discussed the difference between patients with Aspergillus and Candida infection. More articles focusing on Mucor and Cryptococcus infections are needed. Further study is warranted, as our research investigated only the mortality and RRT rates in COVID-19 patients, and more variables should be evaluated.

Our findings suggest that the mechanism by which COVID-19 increases the risk of mycotic infection should be explored further. Our next step is to assess the efficacy of different antifungal medicines in the treatment of COVID-19 patients with fungal coinfection and identify additional criteria to assess the severity of CMI.

## Conclusion

Individuals suffering from both COVID-19 and mycotic infection had higher mortality and RRT rates than those suffering from only COVID-19. The OR for mortality was high in Europe and Asia, where preventive measures should be taken, and more data are needed to fully investigate the situation of CMI. We found Candida spp. infection may lead to a poor outcome, which should be given additional attention. In conclusion, mycotic infection can be regarded as a risk factor for COVID-19. More research into the prevention and treatment of COVID-19 combined with mycotic infection should be conducted.

## Author's note

We define mycotic infection as a risk factor to COVID-19 *via* this meta-analysis and discovered that mortality in EU and Asia is the highest and Candida might be the most dangerous strain of fungi.

## Author contributions

ZL, GS, and AL conceived of and designed the study. ZL and GS had full access to all data, and take responsibility for the integrity of the data and the accuracy of the data analysis, and finished the pathogen detection. YaL, YZ, XC, and JL contributed to the collection of data from the included manuscripts. All authors contributed to data filtration, data analysis, data interpretation and reviewed, and approved the final version of the manuscript.

## Funding

This work was funded by the Guangzhou Institute of Respiratory Health Open Project (Funds provided by the China Evergrande Group) - Project No (2020GIRHHMS14), the Guangzhou Institute of Respiratory Health, Zhongnanshan Medical Foundation of Guangdong Province (ZNSA-2020003 and ZNSA-2020019), Guangdong Basic and Applied Basic Research Foundation (2022A1515010089), and Science and Technology Program of Guangzhou (202201020537).

## Conflict of interest

The authors declare that the research was conducted in the absence of any commercial or financial relationships that could be construed as a potential conflict of interest.

## Publisher's note

All claims expressed in this article are solely those of the authors and do not necessarily represent those of their affiliated organizations, or those of the publisher, the editors and the reviewers. Any product that may be evaluated in this article, or claim that may be made by its manufacturer, is not guaranteed or endorsed by the publisher.

## Author disclaimer

The findings and conclusions in this report are those of the authors' and do not necessarily represent the official position of the Centers for Disease Control and Prevention (CDC). The authors alone are responsible for the content and the writing of the paper.

## References

[1] (JHU) T.C.f.S.S.a.E.C.a.J.H.U. COVID-19 Dashboard. Johns Hopkins University (JHU). Available online at: https://coronavirus.jhu.edu/map.html

[2] Tali SHS LeBlan JJ Nikpour B Armanfard N Sadiq Z Sagan SM. Tools and techniques for severe acute respiratory syndrome__coronavirus 2 (SARS-CoV-2)/COVID-19 detection. Clin Microbiol Rev. (2021) 34:e00228–20. 10.1128/CMR.00228-20 33980687 PMC8142517

[3] Latgé JP. Aspergillus fumigatus and aspergillosis. Clin Microbiol Rev. (1999) 12:310–50. 10.1128/CMR.12.2.310 10194462 PMC88920

[4] Denham ST Wambaugh MA Brown JCS. How environmental fungi cause a range of clinical outcomes in susceptible hosts. J Mol Biol. (2019) 431:2982–3009. 10.1016/j.jmb.2019.05.003 31078554 PMC6646061

[5] Sun S Hoy MJ Heitman J. Fungal pathogens. Curr Biol. (2020) 30:R1163–r1169. 10.1016/j.cub.2020.07.032 33022261

[6] Ledoux MP Guffroy B Nivoix Y Simand C Herbrecht R. Invasive pulmonary aspergillosis. Semin Respir Crit Care Med. (2020) 41:80–98. 10.1055/s-0039-3401990 32000286

[7] Kousha M Tadi R Soubani AO. Pulmonary aspergillosis: a clinical review. Eur Respir Rev. (2011) 20:156–74. 10.1183/09059180.00001011 21881144 PMC9584108

[8] Gonzalez-Lara MF Ostrosky-Zeichner L. Invasive candidiasis. Semin Respir Crit Care Med. (2020) 41:3–12. 10.1055/s-0040-1701215 32000280

[9] Ostrosky-Zeichner L Al-Obaidi M. Invasive fungal infections in the intensive care unit. Infect Dis Clin N Am. (2017) 31:475–87. 10.1016/j.idc.2017.05.005 28687215

[10] Steinbrink JM Miceli MH. Mucormycosis. Infect Dis Clin N Am. (2021) 35:435–52. 10.1016/j.idc.2021.03.009 34016285 PMC10110349

[11] Rawson TM Moore LSP Zhu N Ranganathan N Skolimowska K Gilchrist M. Bacterial and fungal coinfection in individuals with coronavirus: a rapid review to support COVID-19 antimicrobial prescribing. Clin Infect Dis. (2020) 71:2459–68. 10.1093/cid/ciaa530 32358954 PMC7197596

[12] Apostolopoulou A Esquer Garrigos Z Vijayvargiya P Lerner AH Farmakiotis D. Invasive pulmonary aspergillosis in patients with SARS-CoV-2 infection: a systematic review of the literature. Diagnostics. (2020) 10:807. 10.3390/diagnostics10100807 33050499 PMC7600775

[13] Garcia-Vidal C Sanjuan G Moreno-García E Puerta-Alcalde P Garcia-Pouton N Chumbita M. Incidence of co-infections and superinfections in hospitalized patients with COVID-19: a retrospective cohort study. Clin Microbiol Infect. (2021) 27:83–8. 10.1016/j.cmi.2020.07.041 32745596 PMC7836762

[14] Lamoth F Glampedakis E Boillat-Blanco N Oddo M Pagani JL. Incidence of invasive pulmonary aspergillosis among critically ill COVID-19 patients. Clin Microbiol Infect. (2020) 26:1706–8. 10.1016/j.cmi.2020.07.010 32659385 PMC7348600

[15] Singh S Verma N Kanaujia R Chakrabarti A Rudramurthy SM. Mortality in critically ill patients with coronavirus disease 2019-associated pulmonary aspergillosis: a systematic review and meta-analysis. Mycoses. (2021) 64:1015–27. 10.1111/myc.13328 34057252

[16] Chong WH Neu KP. Incidence, diagnosis and outcomes of COVID-19-associated pulmonary aspergillosis (CAPA): a systematic review. J Hosp Infect. (2021) 113:115–29. 10.1016/j.jhin.2021.04.012 33891985 PMC8057923

[17] Lai CC Yu WL. COVID-19 associated with pulmonary aspergillosis: a literature review. J Microbiol Immunol Infect. (2021) 54:46–53. 10.1016/j.jmii.2020.09.004 33012653 PMC7513876

[18] Rawson TM Wilson RC Holmes A. Understanding the role of bacterial and fungal infection in COVID-19. Clin Microbiol Infect. (2021) 27:9–11. 10.1016/j.cmi.2020.09.025 32979569 PMC7546203

[19] Yusuf E Seghers L Hoek RAS van den Akker JPC Bode LGM Rijnders BJA. Aspergillus in critically ill COVID-19 patients: a scoping review. J Clin Med. (2021) 10:2469. 10.3390/jcm10112469 34199528 PMC8215643

[20] Mitaka H Kuno T Takagi H Patrawalla P. Incidence and mortality of COVID-19-associated pulmonary aspergillosis: a systematic review and meta-analysis. Mycoses. (2021) 64:993–1001. 10.1111/myc.13292 33896063 PMC8251156

[21] Chong WH Saha BK Neu KP. Comparing the clinical characteristics and outcomes of COVID-19-associate pulmonary aspergillosis (CAPA): a systematic review and meta-analysis. Infection. (2021) 1–14. 10.1007/s15010-021-01701-x 34570355 PMC8475405

[22] Sinha A Bhaskar SMM. In-hospital prevalence of mucormycosis among coronavirus disease 2019 (COVID-19) patients and COVID-19 in mucormycosis: a systematic review and meta-analysis. Int Forum Allergy Rhinol. (2021) 12:313–317. 10.1002/alr.22906 34633150 PMC8652882

[23] Segrelles-Calvo G de S Araújo GR Llopis-Pastor E Carrillo J Hernández-Hernández M Rey L. Candida spp. co-infection in COVID-19 patients with severe pneumonia: Prevalence study and associated risk factors. Respir Med. (2021) 188:106619. 10.1016/j.rmed.2021.106619 34555702 PMC8445759

[24] Pemán J Ruiz-Gaitán A García-Vidal C Salavert M Ramírez P Puchades F. Fungal co-infection in COVID-19 patients: Should we be concerned? Rev Iberoam Micol. (2020) 37:41–6. 10.1016/j.riam.2020.07.001 33041191 PMC7489924

[25] Tandukar S Palevsky PM. Continuous renal replacement therapy: who, when, why, and how. Chest. (2019) 155:626–38. 10.1016/j.chest.2018.09.004 30266628 PMC6435902

[26] Yang X Tian S Guo H. Acute kidney injury and renal replacement therapy in COVID-19 patients: a systematic review and meta-analysis. Int Immunopharmacol. (2021) 90:107159. 10.1016/j.intimp.2020.107159 33223467 PMC7608016

[27] Horby P Lim WS Emberson JR Mafham M Bell JL Linsell L. Dexamethasone in hospitalized patients with Covid-19. N Engl J Med. (2021) 384:693–704. 10.1056/NEJMoa2021436 32678530 PMC7383595

[28] Sterne JAC Murthy S Diaz JV Slutsky AS Villar J Angus DC. Association between administration of systemic corticosteroids and mortality among critically ill patients with COVID-19: a meta-analysis. JAMA. (2020) 324:1330–41. 10.1001/jama.2020.17023 32876694 PMC7489434

[29] Calzetta L Aiello M Frizzelli A Rogliani P Chetta A. Dexamethasone in patients hospitalized with COVID-19: whether, when and to whom. J Clin Med. (2021) 10:1607. 10.1183/13993003.congress-2021.PA815 33920093 PMC8069656

[30] Das S Rastogi A Harikumar KVS Dutta D Sahay R Kalra S. Diagnosis and management considerations in steroid-related hyperglycemia in COVID-19: a position statement from the endocrine society of India. Indian J Endocrinol Metab. (2021) 25:4–11. 10.4103/ijem.ijem_227_21 34386386 PMC8323636

[31] Barnes BJ Adrover JM Baxter-Stoltzfus A Borczuk A Cools-Lartigue J Crawford JM. Targeting potential drivers of COVID-19: neutrophil extracellular traps. J Exp Med. (2020) 217:e20200652. 10.1084/jem.20200652 32302401 PMC7161085

[32] Borges L Pithon-Curi TC Curi R Hatanaka E. COVID-19 and neutrophils: the relationship between hyperinflammation and neutrophil extracellular traps. Mediators Inflamm. (2020) 2020:8829674. 10.1155/2020/8829674 33343232 PMC7732408

[33] Al-Tawfiq JA Alhumaid S Alshukairi AN Temsah MH Barry M Al Mutair A. COVID-19 and mucormycosis superinfection: the perfect storm. Infection. (2021) 49:833–53. 10.1007/s15010-021-01670-1 34302291 PMC8302461

[34] Fernandes KE Carter DA. Cellular plasticity of pathogenic fungi during infection. PLoS Pathog. (2020) 16:e1008571. 10.1371/journal.ppat.1008571 32497133 PMC7271979

[35] Bartoletti M. Epidemiology of invasive pulmonary aspergillosis among COVID-19 intubated patients: a prospective study. Clin Infect Dis. (2020) 73:e3606–e3614. 10.1093/cid/ciaa1065 32719848 PMC7454393

[36] Ghazanfari M Arastehfar A Davoodi L Yazdani Charati J Moazeni M Abastabar M. Pervasive but neglected: a perspective on COVID-19-associated pulmonary mold infections among mechanically ventilated COVID-19 patients. Front Med. (2021) 8:649675. 10.3389/fmed.2021.649675 34195207 PMC8236642

[37] Hatzl S Reisinger AC Posch F Prattes J Stradner M Pilz S. Antifungal prophylaxis for prevention of COVID-19-associated pulmonary aspergillosis in critically ill patients: an observational study. Crit Care. (2021) 25:335. 10.1186/s13054-021-03753-9 34526087 PMC8441945

[38] Prestel C Anderson E Forsberg K Lyman M de Perio MA Kuhar D. Candida auris outbreak in a COVID-19 specialty care unit - Florida, July-August 2020. MMWR Morb Mortal Wkly Rep. (2021) 70:56–7. 10.15585/mmwr.mm7002e3 33444298 PMC7808709

[39] Singh AK Singh R Joshi SR Misra A. Mucormycosis in COVID-19: A systematic review of cases reported worldwide and in India. Diabetes Metab Syndr. (2021) 15:102146. 10.1016/j.dsx.2021.05.019 34192610 PMC8137376

