# Supplementary material for: Mycotic infection as a risk factor for COVID-19: A meta-analysis
Source: Front Public Health. 2022 Sep 7;10:943234. doi: 10.3389/fpubh.2022.943234 (PMC9489839; doi:10.3389/fpubh.2022.943234)
Supplement: Supplementary file 1 [file Data_Sheet_1.docx]

**Supplementary Materials**

**Contents**

**Supplementary Methods**

Detailed search strategy…………………………………………………….…………1

**Supplementary Tables**

Table 1…………………………………………………………………………………9

Table 2…………………………………………………………………….……….....10

Table 3 ……………………………………………………………………...…..........14

**Supplementary Figure**

Figure 1………………………….……………….………………………...…...........16

**Supplementary Methods**

**Detailed search strategy**

1. PubMed strategy

**#1**

"covid 19"[MeSH Terms] OR ("covid 19"[Title/Abstract] OR "covid 19 virus disease*"[Title/Abstract] OR "disease covid 19 virus"[Title/Abstract] OR "virus disease covid 19"[Title/Abstract] OR "covid 19 virus infection*"[Title/Abstract] OR (("infect"[All Fields] OR "infectability"[All Fields] OR "infectable"[All Fields] OR "infectant"[All Fields] OR "infectants"[All Fields] OR "infected"[All Fields] OR "infecteds"[All Fields] OR "infectibility"[All Fields] OR "infectible"[All Fields] OR "infecting"[All Fields] OR "infections"[All Fields] OR "infections"[MeSH Terms] OR "infections"[All Fields] OR "Infection"[All Fields] OR "infective"[All Fields] OR "infectiveness"[All Fields] OR "infectives"[All Fields] OR "infectivities"[All Fields] OR "infects"[All Fields] OR "pathogenicity"[MeSH Subheading] OR "pathogenicity"[All Fields] OR "infectivity"[All Fields]) AND "covid 19 virus"[Title/Abstract]) OR "virus infection covid 19"[Title/Abstract] OR "2019 ncov infection*"[Title/Abstract] OR "infection 2019 ncov"[Title/Abstract] OR "coronavirus disease 19"[Title/Abstract] OR "2019 novel coronavirus disease"[Title/Abstract] OR "2019 novel coronavirus infection"[Title/Abstract] OR "2019 ncov disease*"[Title/Abstract] OR "coronavirus disease 2019"[Title/Abstract] OR "disease 2019 coronavirus"[Title/Abstract] OR "sars coronavirus 2 infection"[Title/Abstract] OR "sars cov 2 infection"[Title/Abstract] OR "infection sars cov 2"[Title/Abstract] OR "sars cov 2 infection*"[Title/Abstract] OR "covid 19 pandemic*"[Title/Abstract] OR "pandemic covid 19"[Title/Abstract]) OR ("sars cov 2"[MeSH Terms] OR ("coronavirus disease 2019 virus"[Title/Abstract] OR "2019 novel coronavirus*"[Title/Abstract] OR "coronavirus 2019 novel"[Title/Abstract] OR "wuhan seafood market pneumonia virus"[Title/Abstract] OR "novel coronavirus 2019"[Title/Abstract] OR "sars cov 2 virus*"[Title/Abstract] OR "virus sars cov 2"[Title/Abstract] OR "2019 ncov"[Title/Abstract] OR "covid 19 virus*"[Title/Abstract] OR "virus covid 19"[Title/Abstract] OR "wuhan coronavirus"[Title/Abstract] OR "sars coronavirus 2"[Title/Abstract] OR "coronavirus 2 sars"[Title/Abstract] OR "severe acute respiratory syndrome coronavirus 2"[Title/Abstract]))

**#2**

("sars cov 2"[MeSH Terms] AND "SARS-CoV-2 variants"[Supplementary Concept]) OR ("covid 19 virus variants"[Title/Abstract] OR (("sars cov 2"[MeSH Terms] OR "sars cov 2"[All Fields] OR "sars cov 2"[All Fields]) AND "P.2"[Title/Abstract]) OR ("zeta"[All Fields] AND "variant sars cov 2"[Title/Abstract]) OR ((("sars cov 2"[MeSH Terms] OR "sars cov 2"[All Fields] OR "sars cov 2"[All Fields]) AND "20B"[All Fields]) AND "variant"[Title/Abstract]) OR (("P.2"[Supplementary Concept] OR "P.2"[All Fields] OR "P.2"[All Fields]) AND "sars cov 2 variant"[Title/Abstract]) OR ((("sars cov 2"[MeSH Terms] OR "sars cov 2"[All Fields] OR "sars cov 2"[All Fields]) AND ("variant"[All Fields] OR "variant s"[All Fields] OR "variants"[All Fields])) AND "P.2"[Title/Abstract]) OR "sars cov 2 p 1*"[Title/Abstract] OR (("gamma rays"[MeSH Terms] OR ("gamma"[All Fields] AND "rays"[All Fields]) OR "gamma rays"[All Fields] OR "gamma"[All Fields] OR "gamma s"[All Fields] OR "gammae"[All Fields] OR "gammas"[All Fields]) AND "variant sars cov 2"[Title/Abstract]) OR "20j 501y v3"[Title/Abstract] OR "sars cov 2 variant p 1"[Title/Abstract] OR "sars cov 2 cluster 5*"[Title/Abstract] OR "sars cov 2 variant cluster 5"[Title/Abstract] OR ((("sars cov 2"[MeSH Terms] OR "sars cov 2"[All Fields] OR "sars cov 2"[All Fields]) AND "mink-associated"[All Fields]) AND "variant"[Title/Abstract]) OR "cluster 5 sars cov 2 variant"[Title/Abstract] OR (("SARS-CoV-2 variants"[Supplementary Concept] OR "SARS-CoV-2 variants"[All Fields]) AND "variant"[Title/Abstract]) OR (("sars cov 2"[MeSH Terms] OR "sars cov 2"[All Fields] OR "sars cov 2"[All Fields]) AND "A.1.177"[Title/Abstract]) OR (("SARS-CoV-2 variants"[Supplementary Concept] OR "SARS-CoV-2 variants"[All Fields]) AND "variant"[Title/Abstract]) OR (("SARS-CoV-2 variants"[Supplementary Concept] OR "SARS-CoV-2 variants"[All Fields]) AND "variant"[Title/Abstract]) OR "sars cov 2 b 1 429"[Title/Abstract] OR ("B.1.429"[All Fields] AND "sars cov 2 variant"[Title/Abstract]) OR (("sars cov 2"[MeSH Terms] OR "sars cov 2"[All Fields] OR "sars cov 2"[All Fields]) AND "epsilon variant"[Title/Abstract]) OR "sars cov 2 b 1 42*"[Title/Abstract] OR (("sars cov 2"[MeSH Terms] OR "sars cov 2"[All Fields] OR "sars cov 2"[All Fields]) AND "b 1 617 2 variant"[Title/Abstract]) OR (("delta"[All Fields] OR "deltae"[All Fields] OR "deltaes"[All Fields] OR "deltas"[All Fields]) AND "variant sars cov 2"[Title/Abstract]) OR ("B.1.617.2"[All Fields] AND "sars cov 2 variant"[Title/Abstract]) OR (("sars cov 2"[MeSH Terms] OR "sars cov 2"[All Fields] OR "sars cov 2"[All Fields]) AND "variant*"[Title/Abstract]) OR ((("sars cov 2"[MeSH Terms] OR "sars cov 2"[All Fields] OR "sars cov 2"[All Fields]) AND "P.3"[All Fields]) AND "variant"[Title/Abstract]) OR ((("sars cov 2"[MeSH Terms] OR "sars cov 2"[All Fields] OR "sars cov 2"[All Fields]) AND "20B"[All Fields] AND "S265C"[All Fields]) AND "variant"[Title/Abstract]) OR ((("sars cov 2"[MeSH Terms] OR "sars cov 2"[All Fields] OR "sars cov 2"[All Fields]) AND ("theta"[All Fields] OR "thetas"[All Fields])) AND "variant"[Title/Abstract]) OR "sars cov 2 b 1 526 variant"[Title/Abstract] OR ("iota"[All Fields] AND "variant sars cov 2"[Title/Abstract]) OR ("B.1.526"[All Fields] AND "sars cov 2 variant"[Title/Abstract]) OR ((("sars cov 2"[MeSH Terms] OR "sars cov 2"[All Fields] OR "sars cov 2"[All Fields]) AND "iota"[All Fields]) AND "variant"[Title/Abstract]) OR "sars cov 2 b 1 617 1 variant"[Title/Abstract] OR (("kappa"[All Fields] OR "kappa s"[All Fields] OR "kappas"[All Fields]) AND "variant sars cov 2"[Title/Abstract]) OR ((("sars cov 2"[MeSH Terms] OR "sars cov 2"[All Fields] OR "sars cov 2"[All Fields]) AND "21A"[All Fields]) AND "variant"[Title/Abstract]) OR "sars cov 2 b 1 617 1"[Title/Abstract] OR (("SARS-CoV-2 variants"[Supplementary Concept] OR "SARS-CoV-2 variants"[All Fields]) AND "variant"[Title/Abstract]) OR ((("sars cov 2"[MeSH Terms] OR "sars cov 2"[All Fields] OR "sars cov 2"[All Fields]) AND "452R"[All Fields]) AND "variant"[Title/Abstract]) OR "cal 20c*"[Title/Abstract] OR "sars cov 2 b 1 525 variant"[Title/Abstract] OR ("eta"[All Fields] AND "variant sars cov 2"[Title/Abstract]) OR ((("sars cov 2"[MeSH Terms] OR "sars cov 2"[All Fields] OR "sars cov 2"[All Fields]) AND "20A"[All Fields]) AND "variant"[Title/Abstract]) OR ("B.1.525"[All Fields] AND "sars cov 2"[Title/Abstract]) OR "sars cov 2 a 23 1*"[Title/Abstract] OR "sars cov 2 b 1 351 variant"[Title/Abstract] OR ((("sars cov 2"[MeSH Terms] OR "sars cov 2"[All Fields] OR "sars cov 2"[All Fields]) AND ("variant"[All Fields] OR "variant s"[All Fields] OR "variants"[All Fields])) AND "20H-501Y.V2"[Title/Abstract]) OR "sars cov 2 501y v2 variant"[Title/Abstract] OR (("beta"[Journal] OR "beta"[All Fields]) AND "variant sars cov 2"[Title/Abstract]) OR "501y v2 sars cov 2 variant"[Title/Abstract] OR "20H-501Y.V2"[Title/Abstract] OR "sars cov 2 beta variant"[Title/Abstract] OR ((("sars cov 2"[MeSH Terms] OR "sars cov 2"[All Fields] OR "sars cov 2"[All Fields]) AND ("variant"[All Fields] OR "variant s"[All Fields] OR "variants"[All Fields])) AND "20H-501Y.V2"[Title/Abstract]) OR "sars cov 2 d614g variant"[Title/Abstract] OR (("sars cov 2"[MeSH Terms] OR "sars cov 2"[All Fields] OR "sars cov 2"[All Fields]) AND "614g*"[Title/Abstract]) OR ((("sars cov 2"[MeSH Terms] OR "sars cov 2"[All Fields] OR "sars cov 2"[All Fields]) AND "C.37"[All Fields]) AND "variant"[Title/Abstract]) OR (("lambda"[All Fields] OR "lambda s"[All Fields] OR "lambdae"[All Fields] OR "lambdas"[All Fields]) AND "variant sars cov 2"[Title/Abstract]) OR (("sars cov 2"[MeSH Terms] OR "sars cov 2"[All Fields] OR "sars cov 2"[All Fields]) AND "lambda variant"[Title/Abstract]) OR "sars cov 2 b 1 1 7 variant"[Title/Abstract] OR (("alpha"[All Fields] OR "alpha s"[All Fields] OR "alphas"[All Fields]) AND "variant sars cov 2"[Title/Abstract]) OR (("sars cov 2"[MeSH Terms] OR "sars cov 2"[All Fields] OR "sars coronavirus 2"[All Fields]) AND "variant vui 202012 01"[Title/Abstract]) OR ((("sars cov 2"[MeSH Terms] OR "sars cov 2"[All Fields] OR "severe acute respiratory syndrome coronavirus 2"[All Fields]) AND ("variant"[All Fields] OR "variant s"[All Fields] OR "variants"[All Fields])) AND "202012-01"[Title/Abstract]) OR "sars cov 2 voc 202012 01"[Title/Abstract] OR "sars cov 2 b 1 1 7"[Title/Abstract] OR "sars cov 2 variant of concern 202012 01"[Title/Abstract] OR "sars cov 2 20i 501y v1"[Title/Abstract] OR "20I-501Y.V1"[Title/Abstract] OR "sars cov 2 alpha variant"[Title/Abstract] OR "sars cov 2 vui 202012 01"[Title/Abstract])

**#3= #1 OR #2**

**#4**

"lung diseases, fungal"[MeSH Terms] OR ("fungal lung disease"[Title/Abstract] OR "pulmonary fungal diseases"[Title/Abstract] OR ((("fungals"[All Fields] OR "microbiology"[MeSH Terms] OR "microbiology"[All Fields] OR "Fungal"[All Fields] OR "fungi"[MeSH Terms] OR "fungi"[All Fields]) AND "disease*"[All Fields]) AND "Pulmonary"[Title/Abstract]) OR "pulmonary fungal disease"[Title/Abstract] OR "pulmonary fungal infections"[Title/Abstract] OR ((("fungals"[All Fields] OR "microbiology"[MeSH Terms] OR "microbiology"[All Fields] OR "Fungal"[All Fields] OR "fungi"[MeSH Terms] OR "fungi"[All Fields]) AND "infection*"[All Fields]) AND "Pulmonary"[Title/Abstract]) OR "pulmonary fungal infection"[Title/Abstract] OR "fungal lung diseases"[Title/Abstract])

**#5**

"Blastomycosis"[MeSH Terms] OR ("Blastomycoses"[Title/Abstract] OR "blastomycosis north american"[Title/Abstract] OR ("gilchrist*"[All Fields] AND "Disease"[Title/Abstract]))

**#6**

"pneumonia, pneumocystis"[MeSH Terms] OR ("pneumocystis pneumonias"[Title/Abstract] OR "pcp infection*"[Title/Abstract] OR "pneumocystis jirovecii pneumonia"[Title/Abstract] OR "pneumocystos*"[Title/Abstract] OR "p carinii pneumonia*"[Title/Abstract] OR "pneumonia p carinii"[Title/Abstract] OR "pneumocystis carinii pneumonia"[Title/Abstract] OR "pneumonia pneumocystis carinii"[Title/Abstract] OR "p carinii pneumonia*"[Title/Abstract] OR "pneumonia p carinii"[Title/Abstract] OR "p jirovecii pneumonia*"[Title/Abstract] OR "pneumonia p jirovecii"[Title/Abstract] OR "pcp pneumonia*"[Title/Abstract] OR ("pneumonia*"[All Fields] AND "PCP"[Title/Abstract]) OR "pneumonia interstitial plasma cell"[Title/Abstract])

**#7**

（"Pulmonary Aspergillosis"[MeSH Terms] OR (("Pulmonary Aspergillosis"[MeSH Terms] OR ("pulmonary"[All Fields] AND "aspergillosis"[All Fields]) OR "Pulmonary Aspergillosis"[All Fields] OR ("aspergillosis"[All Fields] AND "pulmonary"[All Fields]) OR "aspergillosis pulmonary"[All Fields]) AND ("Pulmonary Aspergillosis"[MeSH Terms] OR ("pulmonary"[All Fields] AND "aspergillosis"[All Fields]) OR "Pulmonary Aspergillosis"[All Fields] OR ("lung"[All Fields] AND "aspergillosis"[All Fields]) OR "lung aspergillosis"[All Fields]) AND ("aspergillos*"[All Fields] AND ("lung"[MeSH Terms] OR "lung"[All Fields])) AND ("Bronchopulmonary"[All Fields] AND "aspergillosis*"[All Fields]) AND ("aspergillose*"[All Fields] AND "Bronchopulmonary"[All Fields]) AND ("Pulmonary Aspergillosis"[MeSH Terms] OR ("pulmonary"[All Fields] AND "aspergillosis"[All Fields]) OR "Pulmonary Aspergillosis"[All Fields] OR ("aspergillosis"[All Fields] AND "Bronchopulmonary"[All Fields])))）

**#8**

"aspergillosis, allergic bronchopulmonary"[MeSH Terms] OR (("Aspergillosis"[MeSH Terms] OR "Aspergillosis"[All Fields] OR "Aspergilloses"[All Fields]) AND "allergic bronchopulmonary"[Title/Abstract]) OR "allergic bronchopulmonary aspergillosis"[Title/Abstract] OR (("Bronchopulmonary"[All Fields] AND "aspergillos*"[All Fields]) AND "Allergic"[Title/Abstract]) OR "allergic bronchopulmonary aspergilloses"[Title/Abstract] OR (("Aspergillosis"[MeSH Terms] OR "Aspergillosis"[All Fields] OR "Aspergilloses"[All Fields]) AND "bronchopulmonary allergic"[Title/Abstract]) OR ((("Allergic"[All Fields] OR "allergical"[All Fields] OR "allergically"[All Fields] OR "allergics"[All Fields] OR "allergization"[All Fields] OR "allergizing"[All Fields]) AND "aspergillos*"[All Fields]) AND "Bronchopulmonary"[Title/Abstract]) OR (("Aspergillosis"[MeSH Terms] OR "Aspergillosis"[All Fields] OR "Aspergilloses"[All Fields]) AND "bronchopulmonary allergic"[Title/Abstract]) OR "bronchopulmonary allergic aspergillos*"[Title/Abstract]

**#9**

"Candidiasis"[MeSH Terms] OR "Candidiases"[Title/Abstract] OR "candida infection*"[Title/Abstract] OR "infection candida"[Title/Abstract] OR "monilias*"[Title/Abstract]

**#10**

"Cryptococcosis"[MeSH Terms] OR ("Cryptococcoses"[Title/Abstract] OR "cryptococcus infections"[Title/Abstract] OR "torulos*"[Title/Abstract] OR "cryptococcus infection"[Title/Abstract] OR "infection cryptococcus"[Title/Abstract] OR "cryptococcus neoformans infection*"[Title/Abstract] OR "c neoformans infection*"[Title/Abstract] OR "c neoformans infection*"[Title/Abstract] OR "cryptococcus gattii infection*"[Title/Abstract] OR (("infect"[All Fields] OR "infectability"[All Fields] OR "infectable"[All Fields] OR "infectant"[All Fields] OR "infectants"[All Fields] OR "infected"[All Fields] OR "infecteds"[All Fields] OR "infectibility"[All Fields] OR "infectible"[All Fields] OR "infecting"[All Fields] OR "infection s"[All Fields] OR "Infections"[MeSH Terms] OR "Infections"[All Fields] OR "Infection"[All Fields] OR "infective"[All Fields] OR "infectiveness"[All Fields] OR "infectives"[All Fields] OR "infectivities"[All Fields] OR "infects"[All Fields] OR "pathogenicity"[MeSH Subheading] OR "pathogenicity"[All Fields] OR "infectivity"[All Fields]) AND "cryptococcus gattii"[Title/Abstract]) OR "c gattii infection*"[Title/Abstract] OR "c gattii infection*"[Title/Abstract] OR ("infection*"[All Fields] AND "c gattii"[Title/Abstract]))

**#11**

"Mucormycosis"[Title/Abstract] OR "mucormycose*"[Title/Abstract] OR "mucorales infection*"[Title/Abstract] OR "Mucor"[Title/Abstract]

1. Web of Science strategy

**#1**

AB=("cohort study" OR "observational study" OR "cohort studies")

**#2**

TI=("cohort study" OR "observational study" OR "cohort studies")

**#3**

#1 OR #2

**#4**

AB=("covid 19" OR "covid 19 virus disease*" OR "disease covid 19 virus" OR "virus disease covid 19" OR "covid 19 virus infection*" OR "2019 novel coronavirus disease" OR "2019 novel coronavirus infection" OR "2019 ncov disease*" OR "coronavirus disease 2019" OR "disease 2019 coronavirus" OR "sars coronavirus 2 infection" OR "sars cov 2 infection" OR "infection sars cov 2" OR "sars cov 2 infection*" OR "covid 19 pandemic*"OR "pandemic covid 19" OR "sars cov 2" OR "coronavirus disease 2019 virus" OR "2019 novel coronavirus*" OR "coronavirus 2019 novel" OR "wuhan seafood market pneumonia virus" OR "novel coronavirus 2019" OR "sars cov 2 virus*"OR "virus sars cov 2" OR "2019 ncov" OR "covid 19 virus*" OR "virus covid 19" OR "wuhan coronavirus" OR "sars coronavirus 2" OR "coronavirus 2 sars" OR "severe acute respiratory syndrome coronavirus 2" )

#5

AB=(("lung diseases, fungal" OR "fungal lung disease" OR "pulmonary fungal diseases" OR "fungals" OR "microbiology" OR "microbiology" OR "Fungal" OR "fungi" OR "fungi" )AND "disease*" AND "Pulmonary" OR "pulmonary fungal disease" OR "pulmonary fungal infections" OR ("fungals" OR "microbiology" OR "microbiology" OR "Fungal" OR "fungi" OR "fungi") AND "infection*" )

**#6**

AB=("Blastomycosis" OR "Blastomycoses" OR "blastomycosis north american" OR ("gilchrist*" AND "Disease"))

**#7**

AB=(("pneumonia, pneumocystis" OR "pneumocystis pneumonias"OR "pcp infection*" OR "pneumocystis jirovecii pneumonia" OR "pneumocystos*" OR "p carinii pneumonia*" OR "pneumonia p carinii" OR "pneumocystis carinii pneumonia" OR "pneumonia pneumocystis carinii" OR "p carinii pneumonia*" OR "pneumonia p carinii" OR "p jirovecii pneumonia*" OR "pneumonia p jirovecii" OR "pcp pneumonia*" OR ("pneumonia*" AND "PCP") OR "pneumonia interstitial plasma cell"))

**#8**

AB=("Pulmonary Aspergillosis" OR ("pulmonary" AND "aspergillosis"[All Fields]) OR ("aspergillosis" AND "pulmonary") OR "aspergillosis pulmonary"OR "Pulmonary Aspergillosis" OR ("lung" AND "aspergillosis") OR "lung aspergillosis" OR ("Bronchopulmonary" AND "aspergillosis*") OR ("aspergillose*" AND "Bronchopulmonary")

**#9**

AB=("aspergillosis, allergic bronchopulmonary" OR (("Aspergillosis" OR "Aspergillosis" OR "Aspergilloses") AND "allergic bronchopulmonary") OR "allergic bronchopulmonary aspergillosis" OR (("Bronchopulmonary" AND "aspergillos*") AND "Allergic") OR "allergic bronchopulmonary aspergilloses" OR (("Aspergillosis" OR "Aspergillosis" OR "Aspergilloses") AND "bronchopulmonary allergic") OR ((("Allergic" OR "allergical" OR "allergically" OR "allergics" OR "allergization" OR "allergizing") AND "aspergillos*") AND "Bronchopulmonary") OR (("Aspergillosis" OR "Aspergillosis" OR "Aspergilloses") AND "bronchopulmonary allergic") OR "bronchopulmonary allergic aspergillos*")

**#10**

AB=("Candidiasis" OR "Candidiases" OR "candida infection*" OR "infection candida" OR "monilias*")

**#11**

AB=(("Cryptococcosis" OR "Cryptococcoses" OR "cryptococcus infections" OR "torulos*" OR "cryptococcus infection" OR "infection cryptococcus" OR "cryptococcus neoformans infection*" OR "c neoformans infection*" OR "c neoformans infection*" OR "cryptococcus gattii infection*" OR (("infect" OR "infectability" OR "infectable" OR "infectant" OR "infectants" OR "infected" OR "infecteds" OR "infectibility" OR "infectible" OR "infecting" OR "infection s" OR "Infections" OR "Infections" OR "Infection" OR "infective" OR "infectiveness" OR "infectives" OR "infectivities" OR "infects" OR "pathogenicity" OR "pathogenicity" OR "infectivity") AND "cryptococcus gattii") OR "c gattii infection*" OR "c gattii infection*" OR ("infection*" AND "c gattii")))

**#12**

AB=("Mucormycosis" OR "mucormycose*" OR "mucorales infection*" OR "Mucor")

**#13**

#3 AND #4

**Supplementary Table 1:** Quality assessment of the included studies

Newcastle-Ottawa Scale for assessing the quality of studies in meta-analysis

| Study | Selection |  |  |  | Comparability control for important factors | Exposure |  |  | Scores |
| --- | --- | --- | --- | --- | --- | --- | --- | --- | --- |
|  | Adequate definition of cases | Representativeness of the cases | Selection of controls | Definition of controls |  | Ascertainment of exposure | Same method of ascertainment for cases and controls | Nonresponse rate |  |
| Alanio.2020 | * | * | * |  | * | * |  |  | 5 |
| Roman-Montes.2021 | * | * | * |  |  |  |  |  | 3 |
| Permpalung.2021 | * | * |  |  | * | * | * | * | 6 |
| Pintado.2021 | * | * |  |  | * | * | * | * | 6 |
| Arkel.2020 | * |  | * |  |  | * | * | * | 5 |
| Bisen.2020 | * | * | * |  | * | * | * | * | 7 |
| Bartolett.2020 | * | * | * |  | * | * | * | * | 7 |
| Lahmer.2021 | * | * |  | * | * | * | * | * | 7 |
| Gangneux.2020 | * | * | * |  | * | * | * | * | 7 |
| Patrucco.2020 | * | * | * |  | * | * | * | * | 7 |
| Fekkar.2020 | * | * | * |  | * | * | * | * | 7 |
| Ghazanfari.2021 | * | * | * |  | * | * | * | * | 7 |
| Segrelles-Calvo.2020 | * | * | * |  | * | * | * | * | 7 |
| Prattes.2021 | * | * | * |  | * | * | * | * | 7 |
| Chauvet.2020 | * | * | * |  | * | * | * | * | 7 |
| Grootveld.2020 | * | * | * |  | * | * | * | * | 7 |
| Delliere.2020 | * | * | * |  | * | * | * | * | 7 |
| Segrelles-Calvo.2021 | * | * | * |  | * | * | * | * | 7 |
| Omrani.2021 | * | * | * |  | * | * | * | * | 7 |
| Gangneux.2021 | * | * | * |  | * | * | * | * | 7 |
| Kumar.2021 | * | * | * |  | * | * | * | * | 7 |

**Supplementary Table 2:** Data extration

| **First Author. year** | **Country** | **Study design: multi/single center-** | **The specie of fungi** | **Mean age** $\boldsymbol{(\pm}\boldsymbol{SD}\mathbf{)}$ | **Mean age control group**$\boldsymbol{(\pm}\boldsymbol{SD}\mathbf{)}$ | **ICU length of stay, mean** | **ICU length of stay, mean (Control group)** | **Mortality** | **Mortality (control group)** | **ECMO rate** | **ECMO rate (Control group)** | **RRT rate** | **RRT rate**  **(Control group)** |
| --- | --- | --- | --- | --- | --- | --- | --- | --- | --- | --- | --- | --- | --- |
| **Pintado.2021(Vélez Pintado et al., 2021)** | **Mexico** | **S** | **Aspergillus** | **64 (10)** | **55 (15)** | **-** | **-** | **31%** | **13%** | **-** | **-** | **-** |  |
| **Segrelles-Calvo.2020(Segrelles-Calvo et al., 2021a)** | **Spain** | **S** | **Aspergillus** | **59.6** | **63** | **32.25 ± 14** | **16.5 ± 10.5** | **86%** | **37%** | **-** | **-** | **-** | **-** |
| **Grootveld.2020(Grootveld, 2020)** | **The Netherlands** | **S** | **Aspergillus** | **65 (59 - 72)** | **61 (55 - 68)** | **-** | **-** | **52.6%** | **20.5%** | **5.3%** | **0%** | **31.6%** | **15.9%** |
| **Lahmer.2021(Lahmer et al., 2021)** | **Germany** | **S** | **Aspergillus** | **-** | **-** | **-** | **-** | **36%** | **9.5%** | **-** | **-** | **55%** | **14%** |
| **Prattes.2021(Prattes et al., 2021)** | **Multinational** | **M** | **Aspergillus** | **-** | **-** | **-** | **-** | **44.0%** | **46.4%** | **7.5%** | **8.3%** | **-** | **-** |
| **Bartoletti.2020(Bartoletti, 2020)** | **Italy** | **M** | **Aspergillus** | **63(7)** | **63(7)** | **16** | **21** | **44%** | **19%** | **-** | **-** | **37%** | **26%** |
| **Fekkar.2020(Fekkar et al., 2021)** | **France** | **S** | **Fungi** | **57.7** | **54.4** | **24** | **30** | **43%** | **24%** | **43%** | **54%** | **57%** | **29%** |
| **Ghazanfari.2020(Ghazanfari et al., 2021)** | **Iran** | **M** | **Fungi** | **-** | **-** | **-** | **-** | **92.5%** | **100%** | **-** | **-** | **-** | **-** |
| **Permpalung.2021(Permpalung, 2021)** | **The U.S.** | **M** | **Aspergillus** | **-** | **-** | **-** | **-** | **56.4%** | **40.3%** | **55.4%** | **14.9%** | **38.5%** | **16.5%** |
| **Patrucco.2021(Patrucco et al., 2021)** | **Italy** | **S** | **Fungi** | **-** | **-** | **15.66** | **16.76** | **16.65** | **4.5%** | **-** | **-** | **-** | **-** |
| **Delliere.2020(Delliere et al., 2020)** | **France** | **M** | **Aspergillus** | **63(56.75-68.25)** | **62(56-68)** | **-** | **-** | **71.4%** | **36.8%** | **4.8%** | **10.3%** | **38.1%** | **34.5%** |
| **Biesen.2020(Van Biesen et al., 2020)** | **The Netherlands** | **S** | **Aspergillus** | **68** | **60** | **37** | **19** | **22.2%** | **15.1%** | **-** | **-** | **-** | **-** |
| **Gangneux.2020(Gangneux et al., 2020)** | **France** | **S** | **Aspergillus** | **70 (63–75)** | **59 (54–68)** | **27** | **12** | **28.6%** | **13.3%** | **-** | **-** | **-** | **-** |
| **Alanio.2020(Alanio, 2021)** | **France** | **S** | **Pneumocystis jirovecii.** | **88** |  | **10** | **20** | **30.0%** | **44.9%** | **10.0%** | **9.2%** | **-** | **-** |
| **Chauvet.2020(Chauvet et al., 2020)** | **France** | **S** | **Aspergillus** | **68(60.7-71.5)** | **66(55.5-70)** | **-** | **-** | **66.7%** | **25%** | **-** | **-** | **-** | **-** |
| **Arkel.2020(van Arkel et al., 2020)** | **The Netherlands** | **S** | **Aspergillus** | **-** | **-** | **-** | **-** | **66.7%** | **32%** | **-** | **-** | **-** | **-** |
| **Roman-Montes**  **.2021(Roman-Montes et al., 2021)** | **Mexico** | **S** | **Aspergillus** | **48.3 (11.7)** | **48.5 (11.5)** | **-** | **-** | **57.1%** | **48.6%** | **-** | **-** | **-** | **-** |
| **Omrani.2021(Omrani et al., 2021)** | **Qatar** | **S** | **Candida** | **61(51-70)** | **52(44-61)** | **-** | **-** | **60%** | **18.1%** | **-** | **-** | **45%** | **22.5%** |
| **Segrelles-Calvo.2021(Segrelles-Calvo et al., 2021b)** | **Spain** | **S** | **Candida** | **62(-)** | **65(-)** | **-** | **-** | **87%** | **36%** | **-** | **-** | **-** | **-** |
| **Gangneux.2021(Gangneux et al., 2021)** | **Multicontinents** | **M** | **Fungi** | **62.0(12.5)** | **58.5(12.4)** | **-** | **-** | **55%** | **30%** | **-** | **-** | **-** | **-** |
| **Kumar.2021(Kumar et al., 2022)** | **India** | **S** | **Mucorales** | **53.6(48.2-59.1)** | **57.2(52.1-62.4)** | **-** | **-** | **73.9%** | **65.4%** | **-** | **-** | **-** | **-** |

S: single-center research; M: multicenter research; ECMO, extracorporeal membrane oxygenation; RRT, renal replacement therapy

**Supplementary Table 3**: Detailed species of *Candida spp.*

| Author | Species | Count |
| --- | --- | --- |
| Omrani(Omrani et al., 2021) |  |  |
|  | *Nakaseomyces glabrata* | 22 |
|  | *C.parapsilosis* | 20 |
|  | *C.albicans* | 18 |
|  | *C.tropicalis* | 11 |
|  | *C.auris* | 7 |
| Segrelles-Calvo(Segrelles-Calvo et al., 2021b) |  |  |
|  | *C.albicans* | 23 |
|  | *C.dubliniensis* | 3 |
|  | *C.glabrata* | 3 |
|  | *C.krusei* | 1 |
|  | *C.parapsilosis* | 10 |
|  | *C.tropicalis* | 1 |

References:

1. Alanio, A. (2021). The presence of Pneumocystis jirovecii in critically ill patients with COVID-19. *J Infect* 82(4)**,** 84-123. doi: 10.1016/j.jinf.2020.11.016.
2. Bartoletti, M. (2020). Epidemiology of invasive pulmonary aspergillosis among COVID-19 intubated patients: a prospective study. *Clinical Infectious Disease*. doi: 10.1093/cid/ciaa1065.
3. Chauvet, P., Mallat, J., Arumadura, C., Vangrunderbeek, N., Dupre, C., Pauquet, P., et al. (2020). Risk Factors for Invasive Pulmonary Aspergillosis in Critically Ill Patients With Coronavirus Disease 2019-Induced Acute Respiratory Distress Syndrome. *Crit Care Explor* 2(11)**,** e0244. doi: 10.1097/CCE.0000000000000244.
4. Delliere, S., Dudoignon, E., Fodil, S., Voicu, S., Collet, M., Oillic, P.A., et al. (2020). Risk factors associated with COVID-19-associated pulmonary aspergillosis in ICU patients: a French multicentric retrospective cohort. *Clin Microbiol Infect*. doi: 10.1016/j.cmi.2020.12.005.
5. Fekkar, A., Lampros, A., Mayaux, J., Poignon, C., Demeret, S., Constantin, J.M., et al. (2021). Occurrence of Invasive Pulmonary Fungal Infections in Patients with Severe COVID-19 Admitted to the ICU. *Am J Respir Crit Care Med* 203(3)**,** 307-317. doi: 10.1164/rccm.202009-3400OC.
6. Gangneux, J.-P., Dannaoui, E., Fekkar, A., Luyt, C.-E., Botterel, F., De Prost, N., et al. (2021). Fungal infections in mechanically ventilated patients with COVID-19 during the first wave: the French multicentre MYCOVID study. *The Lancet Respiratory Medicine*. doi: 10.1016/s2213-2600(21)00442-2.
7. Gangneux, J.P., Reizine, F., Guegan, H., Pinceaux, K., Le Balch, P., Prat, E., et al. (2020). Is the COVID-19 Pandemic a Good Time to Include Aspergillus Molecular Detection to Categorize Aspergillosis in ICU Patients? A Monocentric Experience. *J Fungi (Basel)* 6(3). doi: 10.3390/jof6030105.
8. Ghazanfari, M., Arastehfar, A., Davoodi, L., Yazdani Charati, J., Moazeni, M., Abastabar, M., et al. (2021). Pervasive but Neglected: A Perspective on COVID-19-Associated Pulmonary Mold Infections Among Mechanically Ventilated COVID-19 Patients. *Front Med (Lausanne)* 8**,** 649675. doi: 10.3389/fmed.2021.649675.
9. Grootveld, R.v. (2020). Systematic screening for COVID-19 associated invasive aspergillosis in ICU patients by culture and PCR on tracheal aspirate. *Mycoses*. doi: 10.1111/myc.13259.
10. Kumar, H.M., Sharma, P., Rudramurthy, S.M., Sehgal, I.S., Prasad, K.T., Pannu, A.K., et al. (2022). Serum iron indices in COVID-19-associated mucormycosis: A case-control study. *Mycoses* 65(1)**,** 120-127. doi: 10.1111/myc.13391.
11. Lahmer, T., Kriescher, S., Herner, A., Rothe, K., Spinner, C.D., Schneider, J., et al. (2021). Invasive pulmonary aspergillosis in critically ill patients with severe COVID-19 pneumonia: Results from the prospective AspCOVID-19 study. *PLoS One* 16(3)**,** e0238825. doi: 10.1371/journal.pone.0238825.
12. Omrani, A.S., Koleri, J., Ben Abid, F., Daghfel, J., Odaippurath, T., Peediyakkal, M.Z., et al. (2021). Clinical characteristics and risk factors for COVID-19-associated Candidemia. *Medical Mycology* 59(12)**,** 1262-1266. doi: 10.1093/mmy/myab056.
13. Patrucco, F., Airoldi, C., Falaschi, Z., Bellan, M., Castello, L.M., Filippone, F., et al. (2021). Mycotic infection prevalence among patients undergoing bronchoalveolar lavage with search of SARS-CoV-2 after two negative nasopharyngeal swabs. *J Breath Res* 15(4). doi: 10.1088/1752-7163/ac2290.
14. Permpalung, N. (2021). COVID-19 Associated Pulmonary Aspergillosis in Mechanically Ventilated Patients. *J Microbiol Immunol Infect*.
15. Prattes, J., Wauters, J., Giacobbe, D.R., Salmanton-Garcia, J., Maertens, J., Bourgeois, M., et al. (2021). Risk factors and outcome of pulmonary aspergillosis in critically ill coronavirus disease 2019 patients-a multinational observational study by the European Confederation of Medical Mycology. *Clin Microbiol Infect*. doi: 10.1016/j.cmi.2021.08.014.
16. Roman-Montes, C.M., Martinez-Gamboa, A., Diaz-Lomeli, P., Cervantes-Sanchez, A., Rangel-Cordero, A., Sifuentes-Osornio, J., et al. (2021). Accuracy of galactomannan testing on tracheal aspirates in COVID-19-associated pulmonary aspergillosis. *Mycoses* 64(4)**,** 364-371. doi: 10.1111/myc.13216.
17. Segrelles-Calvo, G., Araujo, G.R.S., Llopis-Pastor, E., Carrillo, J., Hernandez-Hernandez, M., Rey, L., et al. (2021a). Prevalence of opportunistic invasive aspergillosis in COVID-19 patients with severe pneumonia. *Mycoses* 64(2)**,** 144-151. doi: 10.1111/myc.13219.
18. Segrelles-Calvo, G., de, S.A.G.R., Llopis-Pastor, E., Carrillo, J., Hernandez-Hernandez, M., Rey, L., et al. (2021b). Candida spp. co-infection in COVID-19 patients with severe pneumonia: Prevalence study and associated risk factors. *Respir Med* 188**,** 106619. doi: 10.1016/j.rmed.2021.106619.
19. van Arkel, A.L.E., Rijpstra, T.A., Belderbos, H.N.A., van Wijngaarden, P., Verweij, P.E., and Bentvelsen, R.G. (2020). COVID-19-associated Pulmonary Aspergillosis. *Am J Respir Crit Care Med* 202(1)**,** 132-135. doi: 10.1164/rccm.202004-1038LE.
20. Van Biesen, S., Kwa, D., Bosman, R.J., and Juffermans, N.P. (2020). Detection of Invasive Pulmonary Aspergillosis in COVID-19 with Non-directed Bronchoalveolar Lavage. *Am J Respir Crit Care Med*. doi: 10.1164/rccm.202005-2018LE.
21. Vélez Pintado, M., Camiro-Zúñiga, A., Aguilar Soto, M., Cuenca, D., Mercado, M., and Crabtree-Ramirez, B. (2021). COVID-19-associated invasive pulmonary aspergillosis in a tertiary care center in Mexico City. *Med Mycol* 59(8)**,** 828-833. doi: 10.1093/mmy/myab009.

**Supplementary Figure 1:** Figures for Egger’s test and Begg’s test we conducted.

a

b

a. Egger’s publication bias plot, the p value is 0.423; b. Begg’s funnel plot, the p value is 0.423.
